# Prevalence of *G6PD* candidate variants in malaria-endemic populations of northern Brazil

**DOI:** 10.1016/j.bjid.2026.105893

**Published:** 2026-07-08

**Authors:** Leandro Ferreira Lopes Landeira, Daiana de Souza Perce da Silva, Camila de Almeida Velozo, Viviane Cristina Fernandes dos Santos, Karine Oliveira Lima, Marliton Vinicius Pedrosa Evangelista, Rodrigo Medeiros Martorano, Dalma Maria Banic, Cynthia Chester Cardoso

**Affiliations:** a Universidade Federal do Rio de Janeiro (UFRJ), Instituto de Biologia, Laboratório de Virologia Molecular, Cidade Universitária, Rio de Janeiro, RJ, , Brasil; b Instituto Oswaldo Cruz (Fiocruz), Laboratório de Imunologia Clínica, Rio de Janeiro, RJ, Brasil; c Instituto René Rachou (Fiocruz), Plataforma de PCR em tempo real e digital, Belo Horizonte, MG, Brasil; d Universidade Federal do Acre, Laboratório de Doenças Infecciosas da Amazônia Ocidental, Campus Floresta, Cruzeiro do Sul, AC, Brasil

**Keywords:** G6PD deficiency, Polymorphism, Allele frequency, Malaria, Brazil

## Abstract

Glucose-6-Phosphate Dehydrogenase Deficiency (G6PDd) is the most prevalent enzymopathy worldwide and is particularly frequent in malaria-endemic regions. In Brazil, the distribution of G6PD variants is heterogeneous, with predominance of the African A− variant, although regional diversity remains incompletely characterized. This study aimed to genotype seven G6PD variants previously reported in Brazilian populations using a multiplex assay, to determine their frequencies among G6PD Deficient (G6PDd) individuals as well as in a malaria-endemic population from northern Brazil. This study was conducted including two groups from the states of Acre, Amazonas, and Rondônia. The first group comprised 63 males with known G6PD enzymatic status, enabling genotype-phenotype correlation. The second group included 481 individuals from rural communities to estimate population allele frequencies. The multiplex assay successfully genotyped all targeted variants in a single reaction. Among G6PD deficient individuals, 95.8% carried the African A- (376G/202A) variant, confirming its major contribution to G6PDd in this population. In the population-based group, allele frequencies were 7.0% for 376 G and 3.2% for 202A, with higher frequencies observed in Porto Velho, consistent with greater African ancestry. Strong linkage disequilibrium between 376 G and 202A was detected (D′ = 1.0). The remaining variants were rare or absent. No significant association was observed between African G6PD variants and self-reported number of previous malaria episodes in statistical models adjusted for age, area of residence and genetic ancestry. This study demonstrates that multiplex SNaPshot® genotyping is a viable approach for targeted screening of the seven candidate G6PD variants. Despite the predominance of the G6PD A- variant in northern Brazil, regional differences in genetic ancestry likely lead to variable contributions of specific G6PD variants to the deficiency phenotype. Therefore, population-specific genetic surveillance is essential to support safer and more effective malaria treatment strategies in endemic areas.

## Introduction

Glucose-6-Phosphate Dehydrogenase (*G6PD*) is an evolutionarily ancient enzyme, present in virtually all organisms except for those in the Archaea domain, which are mostly anaerobic, and some host-dependent organisms such as *Mycoplasma genitalium* and *Rickettsia prowazekii*.^1^ *G6PD* catalyzes the first step of the pentose phosphate pathway, converting glucose-6-phosphate into ribulose 5-phosphate and producing reduced Nicotinamide Adenine Dinucleotide Phosphate (NADPH) in the process. NADPH acts as a vital reducing agent in numerous biochemical pathways and plays a central role in maintaining cellular redox homeostasis supporting glutathione peroxidase-mediated neutralization of Reactive Oxygen Species (ROS). In erythrocytes, the pentose phosphate pathway represents the primary source of NADPH. Given the constant exposure of these cells to oxidative stress, largely due to the high intracellular concentration of hemoglobin, G6PD plays a pivotal role in sustaining adequate NADPH levels, thereby ensuring the redox balance and overall homeostasis of erythrocytes.^2^

G6PD Deficiency (G6PDd) is an X-linked genetic disorder caused by *G6PD* gene mutations, where hemizygous males and homozygous females are deficient, while heterozygous females exhibit mosaicism due to random X-chromosome inactivation, leading to a spectrum of enzymatic activity and clinical manifestations. These genetic alterations give rise to enzyme variants with different levels of residual activity, associated with a spectrum of biochemical and clinical manifestations including acute hemolytic anemia triggered by oxidative agents (e.g., certain drugs and fava beans), neonatal jaundice, and chronic non-spherocytic hemolytic anemia.^3^^,^^4^

G6PDd is the most prevalent enzymopathy worldwide, affecting over 500 million individuals. Despite its widespread occurrence, the prevalence of the condition differs significantly between populations, ranging from 0% among Native Americans groups to over 20% in certain regions of Africa and Asia.^4^ In Latin America, the highest prevalence of G6PDd was observed in Jamaica, Colombia, Ecuador, and Suriname. In Brazil, prevalence estimates ranged from 0% to 12.9% among men and from 0% to 13.6% among women^5^ and the highest prevalence (16.3%) was observed in Amazonas State.^6^ The largest Brazilian survey of G6PDd conducted to date analyzed approximately 15,000 male individuals from northern Brazil, covering the states of Acre, Amazonas, Amapá, Pará, Roraima, and Rondônia. The average prevalence observed was 5.6%, with regional variations, where Acre had the highest prevalence (8.3%), while Amazonas had the lowest (4%).^7^ Higher frequencies of G6PDd are consistently observed in regions where malaria is or has historically been endemic.^8^^,^^9^ This pattern may be related to a potential protective effect of certain *G6PD* variants against severe *Plasmodium falciparum* infection, although the underlying mechanisms remain under investigation.^10^^,^^11^

Since the first molecular identification of *G6PD* gene variants, an increasing number of mutations have been reported worldwide. To date, over 1,300 *G6PD* variants are documented in population and clinical databases, although nearly two-thirds still lack sufficient evidence for use in the diagnosis of G6PD deficiency.^12^

The *G6PD* African A- variant, prevalent among individuals of sub-Saharan origin, results from two non-synonymous mutations, A376G, known as the A variant, and G202A, known as the Asahi variant. In contrast, the Mediterranean variant (C563T) is predominantly found in the Mediterranean region, particularly in Greece and southern Italy, as well as in Western Asia. The distribution of *G6PD* mutations in Central and Southeast Asia varies greatly among different regions and ethnic populations.^9^

In Brazil, the most frequent variant is A-, accounting for between 12.1% and 99% of G6PD deficiency cases, followed by the Mediterranean, whose prevalence ranges from 1.5% in northern populations (Acre and the Amazonas states) to 5.6% in southern populations (Rio Grande do Sul state).^13^^,^^14^ Other less common variants, such as Seattle (G844C) and Santamaria (A542T and A376G), have also been reported in populations from northern Brazil, along with novel mutations described exclusively in Amazonian populations, such as the Belém (C409T) and Amazônia (C185A).^15^ In fact, about 54% of the G6PDd individuals from a large survey in the Brazilian Amazon region were genotyped as wild-type (non-African and non-Mediterranean variants), suggesting that further screening is required to more accurately elucidate the genetic variations associated to G6PDd in malaria-endemic regions, such as the northern region of Brazil.^7^ Here, we have developed a multiplex system to simultaneously genotype seven candidate *G6PD* variants in subjects from Brazilian North region. Prevalence was determined among G6PD deficient individuals and also randomly in malaria-endemic populations.

## Material and methods

### Participants and study design

This study employed a cross-sectional observational design. Participants from malaria-endemic areas in northern Brazil were recruited for two distinct analyses involving candidate *G6PD* variants.

Group 1 was designed to investigate the genetic basis of the G6PD-deficient phenotype by identifying which *G6PD* variants were associated with reduced enzymatic activity. This group consisted exclusively of adult men (≥ 18-years) from municipalities in the states of Acre (Mâncio Lima, Rodrigues Alves, and Cruzeiro do Sul) and Amazonas (Guajará). These individuals were selected from a cohort of 567 males included in a G6PD deficiency survey. Among them, 24 individuals exhibited reduced enzyme activity and were genotyped to identify the genetic variants associated with *G6PD* deficiency in this population. A comparable subset of 39 individuals with normal enzyme activity was also genotyped and included as a control group.

Group 2 comprised male and female participants from the states of Rondônia (RO) and Amazonas (AM) and was used to estimate the allele frequencies of the seven candidate *G6PD* variants in malaria-endemic populations and their potential association with malaria recurrence. Participants from Amazonas (AM; n = 313) were randomly selected from rural communities in Rio Preto da Eva, located in the metropolitan region of Manaus, in the western Brazilian Amazon, between 2013 and 2015,^16^ while participants from Rondonia (RO; n = 168) were residents of rural communities in Porto Velho.^17^ The populations of these communities comprise both native Amazonian inhabitants and migrants originating from non-endemic regions of Brazil. The study was approved by the Fundação Oswaldo Cruz Ethics Committee (protocol number 346.613 and 354/06).

### G6PD activity test

In males, G6PD deficiency is defined as enzyme activity below 30% of normal.^18^ At this clinically relevant threshold, the qualitative Fluorescent Spot Test (FST, also known as Beutler test) exhibits high diagnostic performance, with reported sensitivities ranging from 90% to 100%.^19^, ^20^, ^21^ For each participant from Group 1, 4 mL of peripheral venous blood was collected into EDTA-containing tubes. Samples were properly labeled and transported to the Laboratory of Infectious Diseases of the Western Amazon, where they were processed and subjected to the FST test. Briefly, 10 µL of whole blood was added to 200 µL of substrate solution and mixed thoroughly. Aliquots of 10 µL were then applied onto filter paper immediately and after 5- and 10-minutes of incubation at 37 °C, generating three sequential spots. After complete drying, the filter paper was examined under Ultraviolet (UV) transillumination by a trained professional to detect the characteristic fluorescence indicative of G6PD activity.

### Variants selection and design of a synthetic *G6PD* fragment

**Fig. 1:**
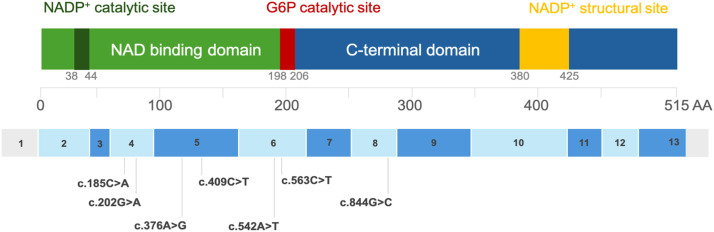
Functional domains of *G6PD* and positions of the analyzed variants. Schematic representation of the G6PD protein structure highlighting functional domains and the analyzed variants positions. Exons are shown as numbered boxes, and the locations of the *G6PD* gene variants analyzed in this study are indicated relative to their corresponding exons and protein domains.

This study focused on seven *G6PD* variants previously reported in Brazilian populations.^5^^,^^22^ The A- (G202A/A376G), A (A376G) and Mediterranean (C563T) variants, the most frequent in Brazil, were selected along with other variants such as Seattle (G844C) and Santamaria (A542T/A376G). Two rare variants, Belém (C409T) and Amazônia (C185A), previously identified in individuals from northern Brazil, were also included due to their regional relevance (Fig. 1). Given the very low population allele frequencies observed for several of the investigated variants, a synthetic fragment containing the seven *G6PD* variants was designed (Supplementary Fig. 1) to verify the analytical performance of the genotyping system in detecting both alleles of each variant.

### DNA isolation

For the first group, peripheral blood samples were collected from the study participants, and DNA was subsequently extracted using the Maxwell® 16 Viral Total Nucleic Acid Purification Kit (Promega, USA), according to the manufacturer’s instructions. Genomic DNA was extracted from peripheral blood samples of participants in the second group, as previously described.^16^^,^^17^

### Multiplex PCR

The genomic regions containing the *G6PD* variants of interest were amplified by multiplex Polymerase Chain Reaction (PCR) using the QIAGEN Multiplex PCR Kit (Qiagen). Four primer pairs were designed to amplify the regions encompassing the seven variants analyzed (Supplementary Table 1). Reactions were performed on a Veriti® 96-Well VeriFlex thermal cycler (Applied Biosystems) under the following conditions: 95 °C for 15-min, followed by 40 cycles of 94 °C for 30 s, 58 °C for 90 s, and 72 °C for 90 s, with a final extension at 72 °C for 90 s. The PCR products were enzymatically purified by adding 1.4 U of Exonuclease I (ExoI) and 3.4 U of Shrimp Alkaline Phosphatase (SAP) to 10 µL of amplicons. Reactions were incubated at 37 °C for 1 h, followed by enzyme inactivation at 75 °C for 15 min.

### SNaPshot® reaction and capillary electrophoresis

A multiplex single-base extension reaction was performed using the SNaPshot® Multiplex Kit and variant-specific primers, each containing a 5′ poly-A tail of distinct length to allow variant discrimination based on fragment size (Supplementary Table 2).

For each reaction, 1.5 μL of the purified PCR product was combined with 2.5 μL of the SNaPshot® Multiplex Kit, 0.5 μL of the primer mix, and 0.5 μL of deionized water, yielding a final reaction volume of 5 μL. Thermal cycling consisted in 30 cycles of 96 °C for 10 s, 50 °C for 5 s, and 60 °C for 30 s, with a ramp rate of 1 °C/s, followed by an additional enzymatic purification step using 1 U of SAP to remove unincorporated ddNTPs. For capillary electrophoresis, 1 μL of the purified extension products was mixed with 8.5 μL of Hi-Di® Formamide (Applied Biosystems) and 0.5 μL of GeneScan® 60 LIZ® size standard (Applied Biosystems). The mixture was denatured at 95 °C for 5-minutes, followed by rapid cooling to 4 °C, and subsequently analyzed on an ABI Prism 3130 Genetic Analyzer (Applied Biosystems). The resulting electropherograms were processed and interpreted using GeneMapper® 4.0 software (Applied Biosystems).

### Genetic ancestry

Ancestry estimates were performed in a previous study (Perce da Silva et al. *Manuscript in preparation*) using a panel of twenty-eight ancestry informative SNPs as described.^23^ Briefly, proportions of African, European and Native American genetic ancestries were estimated using Structure software, version 2.3.1, under an admixture model. Data from European, African and Native American populations from the 1000 Genomes Project were used as reference.^24^ In addition, reference population for Native American ancestry also included individuals from an admixed Amazonian population from Santa Isabel do Rio Negro, all of whom reported recent indigenous ancestry.

### Statistical analysis

Genotypic and allelic frequencies were calculated using PLINK (version 1.9) software. Statistical analyses were performed in RStudio (version 2023.06.0) using the packages “genetics”, “nnet”, and “jmv”. Multinomial logistic regression was applied to evaluate the association between *G6PD* variants and the number of previous malaria episodes, setting category with no previous episodes as reference. To identify the most relevant covariates for inclusion in the multinomial models, a stepwise regression procedure was applied based on the Akaike Information Criterion (AIC) and likelihood ratio test. Linkage Disequilibrium (LD) was assessed for all pairwise combinations of *G6PD* polymorphisms using Haploview (version 4.2) software, based on the Lewontin's (*D'*) coefficient and the *r²* statistic.^25^

## Results

Four fragments containing the seven *G6PD* gene variants were successfully amplified using primers at final concentrations ranging from 2.0 μM to 2.5 μM (Supplementary Table 1). To account for differences in fluorescence peak intensities among the variants, the SNaPshot® multiplex reaction was optimized by adjusting primer concentrations to 1.0‒3.0 μM (Supplementary Table 2), resulting in greater signal homogeneity. This optimization enabled efficient and simultaneous genotyping of the seven selected variants in a single multiplex reaction (Supplementary Fig. 2).

The G6PD deficiency study group (Group 1) comprised 63 male participants, with a mean age of 45.8-years (SD±18.5). Only 7.9% of individuals reported no history of malaria, whereas 27.0% reported a single episode and 65.1% reported between two and five episodes. None of the participants reported more than five malaria episodes. Evaluation of G6PD enzymatic activity revealed alterations in 24 individuals, including three with intermediate activity and 21 with enzymatic deficiency. Normal G6PD activity was observed in the remaining 39 participants. All 63 individuals were successfully genotyped. Among those with altered enzymatic activity, 23 of 24 carried the 376G/202A alleles, consistent with the A- genotype, corresponding to a frequency of 95.8%. As expected, none of the variant alleles were observed among individuals with normal enzymatic activity (Supplementary Table 3).

A total of 481 subjects were enrolled in the investigation of *G6PD* allele frequency and association to malaria recurrence (Group 2). The mean age was 38.1 (±17.2) years, and a slight predominance of males (54.7%) was observed. Native American ancestry was predominant (57.2%±31.2), followed by European (30.8%±27.7) and African ancestries (12.0%±13.2). Regarding malaria history, 18.3% of individuals reported no previous episodes, whereas 44.7% reported up to five malaria episodes and 37.0% reported more than five episodes over their lifetime (Table 1).

**Table 1:** Sociodemographic characteristics, malaria history, genetic ancestry, and distribution of *G6PD* variants among the studied populations.

	Total	Porto Velho	Manaus
	n = 481	n = 168	n = 313
Age (years)			
Mean (SD)	38.1 (17.2)	34.0 (16.5)	40.2 (17.2)
Range	10‒89	12‒85	10‒89
Sex (%)			
Male	263 (54.7)	94 (56.0)	169 (54.0)
Female	218 (45.3)	74 (44.0)	144 (46.0)
Genetic ancestry, mean % (SD)			
European	30.8 (±27.7)	35.9 (±31.7)	29.1 (±26.0)
African	12 (±13.2)	15.1 (±17.2)	10.8 (±11.3)
Native American	57.2 (±31.2)	49 (±33.4)	60.1 (±29.9)
Previous Malaria, n (%)			
0	84 (18.3)	22 (14.7)	62 (20.0)
1	69 (15.0)	21 (14.0)	48 (15.5)
2–5	137 (29.7)	46 (30.7)	91 (29.4)
> 5	170 (37.0)	61 (40.7)	109 (35.2)
*G6PD* variant, n (%)			
Asahi (202A)	0	0	0
A (376 G)	26 (5.4)	13 (7.7)	13 (4.2)
A- (376G/202A)	22 (4.6)	10 (6.0)	12 (3.8)
Amazônia (185A)	0	0	0
Belém (409T)	0	0	0
Santamaria (376G/542T)	0	0	0
Mediterranean (563T)	0	0	0
Seattle (844C)	1 (0.2)	0	1 (0.3)
Undetected^a^	17 (3.5)	17 (10.1)	0
Wild type	415 (86.3)	128 (76.2)	287 (91.7)

a For these samples, incomplete genotyping of one or more *G6PD* SNPs precluded the determination of the corresponding variant.

When stratified by region of origin, differences were observed between participants from Manaus and Porto Velho. Individuals from Porto Velho were younger on average (34±6.5 years) with males accounting for 56% of the sample. Genetic ancestry showed a higher mean contribution of Native American ancestry (49.0%±33.4), followed by European (35.9%±31.7) and African ancestries (15.1%±17.2). Participants from Manaus (n = 313) were older on average (40.2 ± 17.2 years) and display a similar sex distribution (54.0% males), with a higher mean proportion of Native American ancestry (60.1%±29.9) and lower European (29.1%±26.0) and African (10.8%±11.3) contributions. Also, a higher proportion of individuals from Manaus reported no history of malaria (20%), whereas participants from Porto Velho more frequently reported experiencing more than five malaria episodes (40.7%).

Complete genotyping of all seven *G6PD* SNPs was achieved for 464 out of 481 individuals. Twenty-six subjects (5.4%) were found to exclusively carry the African A variant (376 G), while 22 subjects (4.6%) presented the A- variant (376G/202A). The Seattle variant (844C) was detected in a single individual (0.2%). A total of 415 subjects (86.3%) did not exhibit any of the seven variants assessed in this study and were classified as wild type. Regional differences in the distribution of *G6PD* variants were observed, with Porto Velho showing higher frequencies of the A376G (7.7%) and A376G/G202A (6.0%) variants than Manaus (4.2% and 3.8%, respectively).

Despite the predominance of the wild-type allele, the 376 G SNP was the most frequent mutated allele observed in the study population in both sexes. A total of 48 individuals carried the 376 G allele, including 16 hemizygous males, 31 heterozygous females, and one homozygous female, corresponding to an overall allele frequency of 7.0%. The 202A allele was identified in 22 individuals (11 hemizygous males and 11 heterozygous females), yielding a population allele frequency of 3.2%. Sex-stratified analyses revealed a higher frequency of the 202A allele among males (4.2%), whereas the 376 G allele was more prevalent among females (7.6%). The 844C allele was detected only once, in a single heterozygous female participant (0.2%), resulting in an overall allele frequency of 0.1%. No mutations were detected in the remaining *G6PD* variants analyzed (Table 2). Stratification by geographic origin demonstrated higher frequencies of the 376 G and 202A alleles in Porto Velho compared with Manaus. In Porto Velho, both alleles were more frequent in males than females, whereas in Manaus, 376 G was more prevalent among females and 202A among males (Supplementary Table 4). The genotype frequencies of the G202A and A376G SNPs in females were consistent with Hardy-Weinberg equilibrium (p = 0.7024 and p = 0.8096, respectively), while the remaining SNPs could not be assessed as they were monomorphic.

**Table 2:** Genotype and allele distribution of *G6PD* SNPs in Manaus and Porto Velho populations.

Sex	Genotype/ Allele	G202A	A376G	C185A	C409T	A542T	C563T	G844C
Female (n = 218)	1/1	207 (95.0%)	186 (85.3%)	217 (100%)	217 (100%)	214 (100%)	214 (100%)	216 (99.5%)
	1/2	11 (5.0%)	31 (14.2%)	0 (0%)	0 (0%)	0 (0%)	0 (0%)	1 (0.5%)
	2/2	0 (0%)	1 (0.5%)	0 (0%)	0 (0%)	0 (0%)	0 (0%)	0 (0%)
	1	0.975	0.924	1.000	1.000	1.000	1.000	0.998
	2	0.025	0.076	0.000	0.000	0.000	0.000	0.002
Male (n = 260)^a^	1/0	248 (95.8%)	244 (93.8%)	260 (100%)	257 (100%)	252 (100%)	251 (100%)	258 (100%)
	2/0	11 (4.2%)	16 (6.2%)	0 (0%)	0 (0%)	0 (0%)	0 (0%)	0 (0%)
Total (n = 478)	1	0.968	0.930	1.000	1.000	1.000	1.000	0.999
	2	0.032	0.070	0.000	0.000	0.000	0.000	0.001

** 3 samples were removed due to an error between sex and genotype (e.g., heterozygous male).

a Reference and variant alleles were represented as 1 and 2, respectively. Genotypes were represented as 1/0 and 2/0 for male hemizygous. Only male genotypic frequencies are shown, since under hemizygosity they are equivalent to allele frequencies.

**Fig. 2:**
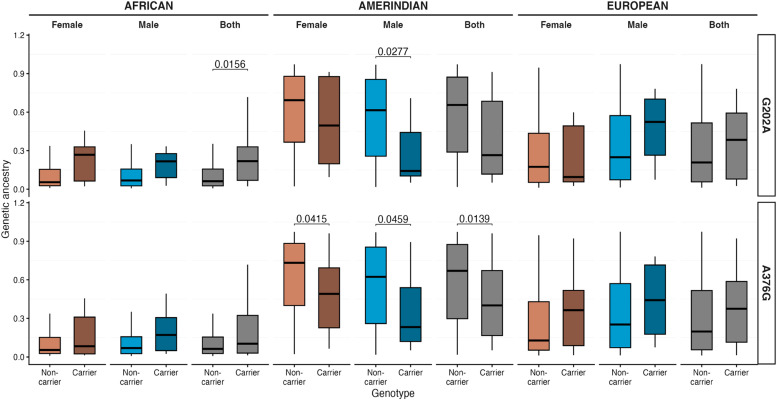
Proportion of genetic ancestry in individuals carrying or not carrying the G202A and A376G alleles, stratified by sex. Individuals with at least one copy of the mutated allele (hemizygous males or heterozygous/homozygous females) were considered carriers, whereas those without any mutated allele were classified as non-carriers. Statistical significance is indicated by p-values above the boxes.

Genetic ancestry proportions were compared between carriers and non-carriers of the *G6PD* G202A and A376G alleles in the overall sample of Group 2 and after stratification by sex (Fig. 2). Carriers of the G202A allele showed a higher proportion of African ancestry than non-carriers when both sexes were analyzed together (p = 0.0156). In addition, male carriers exhibited a lower proportion of Amerindian ancestry compared with non-carriers (p = 0.0277). For the A376G allele, carriers consistently showed a lower proportion of Amerindian ancestry than non-carriers in the overall sample as well as in sex-stratified analyses. No significant differences in the proportion of European ancestry were observed for either variant (Fig. 2).

Linkage disequilibrium analysis was feasible for only three of the seven analyzed *G6PD* SNPs, since the alternative allele was not observed for the remaining markers. Strong linkage disequilibrium was observed between the African-associated SNPs A376G and G202A (*D′* = 1.0; *r^2^* = 0.43) in the overall study population, as well as in the Manaus (*D′* = 1.0; *r^²^* = 0.45) and Porto Velho (*D′* = 1.0; *r^2^* = 0.41) subpopulations (Supplementary Fig. 3). The *D′* values observed in the LD analysis between the G6PD A376G and G202A variants in our population were similar to those reported for several African groups in the 1000 Genomes Project database. However, *r^2^* values showed considerable variability among them. Individuals with African Ancestry in Southwest USA, as well as those from Nigeria and Kenya, presented *r^2^* values similar of those obtained in this study, whereas groups from African Caribbean in Barbados, Gambia, and Sierra Leone showed markedly different values (Supplementary Table 6).

Based on the proposed protective role of *G6PD* variants against malaria, an association analysis was performed between the investigated variants and the number of previous malaria episodes in the allele frequency group. Given the X-linked inheritance of *G6PD*, analyses were stratified by sex. In males, genotypes were coded as wild-type or mutant, whereas in females, due to the low frequency of homozygous mutants (n = 1), genotypes were grouped as wild-type versus variant carriers (heterozygous or homozygous).

A stepwise multinomial regression including age, sex, area of residence, duration of residence in malaria-endemic areas, and genetic ancestry was performed to identify the main confounders associated with previous malaria episodes prior to the genetic association analysis. Age, area of residence, and sex provided the best model fit, yielding the lowest AIC and a significant likelihood ratio test (p < 0.01). An adjusted model including the selected covariates was used to assess the association between *G6PD* polymorphisms and previous malaria episodes. The analysis included only the A376G and G202A SNPs, as the remaining variants were monomorphic in the study population. No significant associations were detected (Supplementary Table 5).

## Discussion

The successful amplification and optimization of the multiplex SNaPshot® assay demonstrate that this approach is a robust and feasible strategy for genotyping *G6PD* SNPs. This technique has previously been demonstrated to be applicable for *G6PD* genotyping,^26^ as well as for other genetic polymorphisms.^27^ The flexibility of the multiplex SNaPshot® assay allows the SNP panel to be readily adapted to include population-specific variants, as demonstrated in other studies, which is particularly relevant in genetically diverse populations with heterogeneous *G6PD* variant profiles. This method is more cost-effective and time-efficient for analyzing predefined SNP panels than Sanger sequencing. However, because it relies on the analysis of predefined targets, rare variants not included in the panel may remain undetected. Furthermore, its applicability is restricted to known variants, and the identification of novel mutations requires sequencing-based approaches.^28^^,^^29^

It is important to note that the G6PD-deficient group consisted exclusively of males with reduced G6PD activity identified by the Beutler fluorescent spot test. Qualitative assays may fail to detect some heterozygous females due to random X-chromosome inactivation, resulting in normal or near-normal enzyme activity despite the presence of pathogenic variants. To minimize this source of phenotype misclassification, genotype-phenotype analyses were restricted to hemizygous males, for whom genotype-phenotype relationships are generally more straightforward.^19^^,^^30^ In this group, the A- (376G/202A) variant was identified in 23 of the 24 participants with altered enzyme activity, reinforcing its predominant role in G6PDd in this population. Only a single individual with altered enzymatic activity did not harbor any of the *G6PD* SNPs investigated, suggesting the possible presence of new or untested variants. Previous studies in Brazilian populations have consistently identified the A- variant as the main genetic determinant of G6PD deficiency, accounting for approximately 12% to 99% of deficient cases.^7^^,^^13^^,^^31^, ^32^, ^33^ Importantly, none of the individuals with normal G6PD enzymatic activity carried any of the analyzed *G6PD* mutations, supporting a genotype-phenotype concordance for the variants included in this panel and underscoring the clinical relevance of targeting the A- haplotype in population-based screening strategies. One of the largest studies of G6PDd individuals reported that African and Mediterranean variants accounted for approximately 45% of cases highlighting the need for broader genetic screening.^7^ In contrast to most studies conducted in Brazilian populations, which typically focus on a limited number of recurrent variants, we implemented a broader and customizable panel of *G6PD* mutations. Although the A- variant was found in 95.8% of enzyme deficient individuals in our cohort, applying this panel to populations with different genetic backgrounds could provide a more comprehensive understanding of *G6PD* diversity.

The strong linkage disequilibrium observed between the African SNPs A376G and G202A in the present study is consistent with previous reports suggesting a limited historical recombination between these loci.^34^^,^^35^ Here, all carriers of the 202A allele also carried 376 G, whereas 376 G frequently occurred independently of 202A. Different correlation between these SNPs have been observed in samples of African origin in the 1000 Genomes Project database^36^ (Supplementary Table 6). In admixed populations such as those studied here, the observed LD pattern may be influenced by the contribution of African ancestry. However, this interpretation should be considered cautiously, as the relatively low frequencies of the 202A and 376 G alleles may also affect LD estimates.

The allele frequency patterns observed for the SNPs 202A and 376 G in the present study closely mirror those reported in large population-based genomic databases, including the 1000 Genomes Project and gnomAD.^36^ Both the 202A allele (3.2%) and the 376 G allele (7.0%) presented frequencies consistent with those reported in genomic reference databases, remaining within the previously described intervals of 0.6%–3.6% and 1.7%–9.0%, respectively. The slightly higher frequency of the 844C allele observed in this study (0.1% compared with < 0.01% in public databases) is likely driven by the presence of a single heterozygous individual, highlighting the influence of stochastic sampling effects that can inflate frequency of rare alleles in relatively small cohorts.

Given their African origin, 202A and 376 G alleles exhibit substantially higher frequencies in populations with African ancestry, while being virtually absent in Native Americans and occurring at low frequencies in European populations.^9^^,^^37^ This ancestry-related gradient is reflected in the present study. In general, individuals carrying the 202A and 376 G alleles exhibited a higher proportion of African genetic ancestry, reaching statistical significance for the 202A allele. In contrast, Amerindian ancestry was observed at lower proportions among carriers of these alleles. This reduction was statistically significant for both 202A and 376 G alleles in males, and for the 376 G allele in females and in the combined sample. Together with the low frequencies of these SNPs reported for Native American and European populations in public genomic databases, these findings further support the notion that non-African ancestry components may contribute to the dilution of G6PD African-derived alleles in highly admixed populations.

The absence of the Mediterranean (C563T) *G6PD* variant in the present study may be explained by its distinct geographic distribution within Brazil. Although this variant is considered one of the most frequent *G6PD* mutations in the country (after the African A and A-), its occurrence is largely concentrated in the Southeast and Southern regions, where historical migration from Mediterranean populations was more intense.^38^^,^^39^ Similarly, the Santamaria (376G/542T), Belém (C409T), and Amazônia (C185A) variants are extremely rare on a global scale and have been reported only once in Brazilian populations.^15^ The absence of these variants in the present cohort is therefore consistent with their very low population frequencies and the limited likelihood of capturing such rare alleles within a moderate sample size. Studies conducted in endemic areas of northern Brazil have reported conflicting results regarding the association between *G6PD* variants and malaria. In Manaus, carriers of the *G6PD* A- and Mediterranean variants exhibited a substantial reduction in the risk of malaria episodes.^40^ Conversely, research involving populations from multiple Amazonian states found an increased frequency of malaria recurrences among G6PDd individuals, while other studies in Rondônia and Acre did not detect significant associations.^7^^,^^32^^,^^41^ In the present study, no association was observed between the African *G6PD* variants (376 G and 202A) and previous malaria episodes. These results were obtained either before or after adjustment for covariates including genetic ancestry, which was not included in previous Brazilian studies. Despite the lack of statistically significant results, the association of the candidate *G6PD* variations cannot be completely ruled out due to the inherent limitations of the cross-sectional study design, the self-reported outcome under investigation and the low frequency of the variants, which may have reduced the statistical power to detect modest genetic effects. Although self-reported malaria history provides valuable information on clinical disease burden, it does not capture the full spectrum of infection, including asymptomatic parasitemia and subclinical cases, which would require active longitudinal surveillance and molecular diagnostic approaches. Importantly, self-reported data are subject to recall bias and may not fully reflect an individual's true lifetime exposure. In addition, we were unable to assess data regarding disease severity. This information would be highly relevant, as previous studies suggest that G6PD deficiency may confer protection against severe *P. falciparum* malaria.^4^^,^^42^^,^^43^ A protective effect against cerebral malaria has been reported in hemizygous males and heterozygous females carrying the A- variant, whereas an increased risk of severe anemia has been described in hemi/homozygous individuals.^44^^,^^45^ However, these associations remain inconsistent across populations, with some studies reporting no significant protective effect.^46^

This study is limited by analyzing only a subset of G6PD variants previously described in Brazilian populations, which may underestimate the genetic diversity related to G6PDd. Here, the genotype-phenotype analyses were restricted to male individuals, as G6PD activity was determined using a qualitative assay. Evaluating genotype-phenotype relationships in heterozygous females is highly relevant for improving our understanding of how lyonization patterns influence phenotypic expression and should be addressed in future studies. Additionally, the lack of enzymatic activity data for the allele frequency group restricted the assessment of the potential functional impact of the detected variants. Incorporating genetic screening for prevalent *G6PD* variants into public health strategies may contribute to safer treatment policies, reduce the risk of drug-induced hemolysis, and support more effective malaria control programs in endemic populations.

## Data availability

The data that support the findings of this study must be requested from the corresponding author.

## Funding

This work was supported by 10.13039/501100004586FAPERJ (E-26/200.511/2023 to CCC and E-26/210.683/2024 to DMB), by FAPAC (PP003–2016/TO009–2018/PT19573112019320000 to RMM), 10.13039/501100003593CNPq (307955/2022–2 to CCC and CNPq-DECIT/404068/2012–0 to DMB).

## Conflicts of interest

The authors declare no conflicts of interest.

## Appendix Supplementary materials

Image, application 1
