# Supplementary material for: Prevalence of G6PD candidate variants in malaria-endemic populations of northern Brazil
Source: Braz J Infect Dis. 2026 Jul 8;30(4):105893. doi: 10.1016/j.bjid.2026.105893 (PMC13356659; doi:10.1016/j.bjid.2026.105893)

BJID-D-26-00098_Supplementary Material

**Supplementary Table 1** Primers used to amplify the genomic regions containing the *G6PD* variants analyzed.

| **Mutation name** | **Orientation** | **Sequences (5'‒3')** | **Size (bp)** | **Concentration (μM)** |
| --- | --- | --- | --- | --- |
| Mediterranean / Santamaria | Forward | AGGTTCTGCACCATCTCCTTGCCCA | 298 | 2.5 |
|  | Reverse | TCATCTGGGAACACAAGGCACGGGA |  |  |
| Belém / Asahi | Forward | CGCTCATAGAGTGGTGGGAGCAC | 231 | 2.0 |
|  | Reverse | GGCCAGTACGATGATGCAGCC |  |  |
| A / Amazônia | Forward | CACTGTGAGGCGGGAACGGG | 166 | 2.0 |
|  | Reverse | AAGTGTGTCCCACCACTGCCC |  |  |
| Seattle | Forward | CTCGTCACAGATGGGCCTGC | 129 | 2.5 |
|  | Reverse | GCAGATGCTGTGTCTGGTGGC |  |  |

**Supplementary Table 2** Primers used for genotyping *G6PD* variants by the SNaPshot^®^ single-base extension assay.

| **Mutation name** | **Nucleotide substitution** | **Sequences (5'‒3') with polyA tail size** | **Size (bp)** | **Concentration (μM)** |
| --- | --- | --- | --- | --- |
| Asahi | c.202G>A | 15(A)GGAACGGGCATAGCCCA | 32 | 3 |
| A | c.376A>G | 20(A)CGCCTCAACAGCCACATG | 38 | 1 |
| Amazônia | c.185C>A | 24(A)GTTCCGGGATGGCCTTCTGC | 44 | 1.5 |
| Belém | c.409C>T | 30(A)GCAAGGCCAGGTAGAAGA | 48 | 1.5 |
| Santamaria | c.542A>T | 35(A)TGTGGTTGGACAGCCGG | 52 | 3 |
| Mediterranean | c.563C>T | 37(A)GTCCTCACGGAACAGGGAG | 56 | 3 |
| Seattle | c.844G>C | 37(A)TACCTTCTCATCACGGACGTCAT | 60 | 2 |

**Supplementary Table 3** Genotype and allele distribution of *G6PD* SNPs in Group 1.

| **Enzymatic activity** | **Genotype/ Allele^a^** | **G202A** | **A376G** | **C185A** | **C409T** | **A542T** | **C563T** | **G844C** |
| --- | --- | --- | --- | --- | --- | --- | --- | --- |
| **Normal** (n = 39) | 1/0 | 39 (100.0%) | 39 (100.0%) | 39 (100.0%) | 39 (100.0%) | 39 (100.0%) | 39 (100.0%) | 39 (100.0%) |
|  | 2/0 | 0 (0.0%) | 0 (0.0%) | 0 (0.0%) | 0 (0.0%) | 0 (0.0%) | 0 (0.0%) | 0 (0.0%) |
| **Altered** (n = 24) | 1/0 | 1 (4.2%) | 1 (4.2%) | 24 (100.0%) | 24 (100.0%) | 24 (100.0%) | 24 (100.0%) | 24 (100.0%) |
|  | 2/0 | 23 (95.8%) | 23 (95.8%) | 0 (0.0%) | 0 (0.0%) | 0 (0.0%) | 0 (0.0%) | 0 (0.0%) |

^a^ Reference and variant alleles were represented as 1 and 2, respectively. Genotypes were represented as 1/0 and 2/0 since all individuals were male hemizygous.

**Supplementary Table 4** Genotype and allele distribution of *G6PD* SNPs in Group 2 by region of origin.

| **Population** | **Sex** | **Genotype/ Allele** | **G202A** | **A376G** | **C185A** | **C409T** | **A542T** | **C563T** | **G844C** |
| --- | --- | --- | --- | --- | --- | --- | --- | --- | --- |
| Porto Velho | Female (n = 74) | 1/1 | 69 (93.2%) | 60 (81.1%) | 73 (100.0%) | 73 (100.0%) | 71 (100.0%) | 71 (100.0%) | 73 (100.0%) |
|  |  | 1/2 | 5 (6.8%) | 14 (18.9%) | 0 (0.0%) | 0 (0.0%) | 0 (0.0%) | 0 (0.0%) | 0 (0.0%) |
|  |  | 2/2 | 0 (0.0%) | 0 (0.0%) | 0 (0.0%) | 0 (0.0%) | 0 (0.0%) | 0 (0.0%) | 0 (0.0%) |
|  |  | 1 | 0.966 | 0.905 | 1.000 | 1.000 | 1.000 | 1.000 | 1.000 |
|  |  | 2 | 0.034 | 0.095 | 0.000 | 0.000 | 0.000 | 0.000 | 0.000 |
|  | Male (n = 91) | 1/0 | 85 (94.4%) | 82 (90.1%) | 91 (100.0%) | 88 (100.0%) | 83 (100.0%) | 82 (100.0%) | 89 (100.0%) |
|  |  | 2/0 | 5 (5.6%) | 9 (9.9%) | 0 (0.0%) | 0 (0.0%) | 0 (0.0%) | 0 (0.0%) | 0 (0.0%) |
|  |  | 1 | 0.944 | 0.901 | 1.000 | 1.000 | 1.000 | 1.000 | 1.000 |
|  |  | 2 | 0.056 | 0.099 | 0.000 | 0.000 | 0.000 | 0.000 | 0.000 |
|  | Total (n = 165) | 1 | 0.958 | 0.904 | 1.000 | 1.000 | 1.000 | 1.000 | 1.000 |
|  |  | 2 | 0.042 | 0.096 | 0.000 | 0.000 | 0.000 | 0.000 | 0.000 |
| Manaus | Female (n = 144) | 1/1 | 138 (95.8%) | 126 (87.5%) | 144 (100.0%) | 144 (100.0%) | 144 (100.0%) | 144 (100.0%) | 143 (99.3%) |
|  |  | 1/2 | 6 (4.2%) | 17 (11.8%) | 0 (0.0%) | 0 (0.0%) | 0 (0.0%) | 0 (0.0%) | 1 (0.7%) |
|  |  | 2/2 | 0 (0.0%) | 1 (0.7%) | 0 (0.0%) | 0 (0.0%) | 0 (0.0%) | 0 (0.0%) | 0 (0.0%) |
|  |  | 1 | 0.979 | 0.934 | 1.000 | 1.000 | 1.000 | 1.000 | 0.997 |
|  |  | 2 | 0.021 | 0.066 | 0.000 | 0.000 | 0.000 | 0.000 | 0.003 |
|  | Male (n = 169) | 1/0 | 163 (96.4%) | 162 (95.9%) | 169 (100.0%) | 169 (100.0%) | 169 (100.0%) | 169 (100.0%) | 169 (100.0%) |
|  |  | 2/0 | 6 (3.6%) | 7 (4.1%) | 0 (0.0%) | 0 (0.0%) | 0 (0.0%) | 0 (0.0%) | 0 (0.0%) |
|  |  | 1 | 0.964 | 0.959 | 1.000 | 1.000 | 1.000 | 1.000 | 1.000 |
|  |  | 2 | 0.036 | 0.041 | 0.000 | 0.000 | 0.000 | 0.000 | 0.000 |
|  | Total (n = 313) | 1 | 0.974 | 0.943 | 1.000 | 1.000 | 1.000 | 1.000 | 0.998 |
|  |  | 2 | 0.026 | 0.057 | 0.000 | 0.000 | 0.000 | 0.000 | 0.002 |

**Supplementary Table 5** Association analysis of *G6PD* variants with the number of previous malaria episodes in Manaus and Porto Velho populations.

| **Variant** | **Previous Malaria, n** | **Female** | | **Male** | |
| --- | --- | --- | --- | --- | --- |
|  |  | **OR (95% CI)^a^** | **p-value^a^** | **OR (95% CI)^a^** | **p-value^a^** |
| 202A^c^ | 1 | 1.31 (0.17–10.18) | 0.796 | ^b^ | ^b^ |
|  | 2–5 | 0.60 (0.08–4.65) | 0.628 | 0.37 (0.06–2.40) | 0.300 |
|  | > 5 | 1.37 (0.22–8.65) | 0.735 | 0.60 (0.13–2.78) | 0.516 |
| 376G^d^ | 1 | 1.55 (0.42–5.71) | 0.507 | 0.26 (0.03–2.42) | 0.396 |
|  | 2–5 | 0.56 (0.15–2.12) | 0.396 | 0.24 (0.04–1.41) | 0.115 |
|  | > 5 | 1.64 (0.51–5.29) | 0.411 | 0.60 (0.16–2.21) | 0.411 |

^a^ Adjusted for age and place of residence.

^b^ Category not analyzed as it contains no individuals.

^c^ 202A genotype includes hemizygous A males and G/A or A/A females.

^d^ 376G genotype includes hemizygous G males and A/G or G/G females.

**Supplementary Table 6** Linkage Disequilibrium (LD) between the G6PD 376G and G202A variants in African populations from the 1000 genomes project.

| **Population** | ***r²*** | ***D'*** |
| --- | --- | --- |
| ACB (African Caribbean in Barbados) | 0.220698 | 0.857705 |
| ASW (African Ancestry in Southwest USA) | 0.497601 | 0.999993 |
| ESN (Esan in Nigeria) | 0.295156 | 0.999991 |
| GWD (Gambian in Western Division) | 0.089252 | 0.999977 |
| LWK (Luhya in Kenya) | 0.447062 | 0.999988 |
| MSL (Mende in Sierra Leone) | 0.193747 | 0.999992 |
| YRI (Yoruba in Ibadan, Nigeria) | 0.423302 | 0.999995 |

**Supplementary Figure 1 gBlock sequence.** Sequence containing the seven *G6PD* variants analyzed, based on the reference sequence NC_000023.11 (GRCh38.p14 Primary Assembly).

AGCTCAGTGCCTCGTCACAGATGGGCCTGCGACAGGGCATGCTCCTGGGGACTGGGGTGCACCCCCTACCTTCTCATCACGGACGTCAT[**C844G**]TGAGTTGGTGGAGGCGGGCTTCTCCATGGCCACCAGACACAGCATCTGCAGTAGGTGGTTCTGCATCACGTCCCTGGGGACGGAAGAGGCCAGAGCTCGCCTTAGCTCCCCGCCCTGTTCACCTGGTTCAAGGGCATGGGGACCCCAAACAAGGCTTCCTAGTGACAAGCTGCAAGACTCACTTCCTGATCCCCCTTGTCTTCCAGGTCCCCTTAAATCAAGGAAGGACATGGTGATGCTATCACTGAATCATAAAACCGTGGGGTGCTTGGCTGTGTAGGGGTCCAGCCCTTTCCAGCCCGGTCTGATAGCTCAGACACTTAGGTTTTGAACTGCAGGGTGAGGAGGAGCTCCCCCAAGATAGGGAAGAGTAGCCCTGCAGGGTGACTGGCTCTGCCACCCTGTGCCAGCCTCCCAGGAGAGAGGAAGAGCTCTCACCGGATGATCCCAAATTCATCGAAATAGCCCCCGCGACCCTCAGTGCCAAAGGGCTCCTTGAAGGTGAGGATAACGCAGGCGATGTTGTCCCGGTTCCAGATGGGGCCGAAGATCCTGTTGGCAAATCTGCAGGGAGGGGCAAGGTGGAGGAACTGACCTTGGGCCTCTGTGGTGCAGGGGCCACATGTGAGGGGTCACCCTTGTCTGAGTTCTGGAGGAATTCGTCCTCGGGGAGGCAGTGGGCCAGGTGAGGCTCCTGAGTACCACCCCCACCCTGGTCCCCCGGCCCAGGCTTGGCCCCACCTCAGCACCATGAGGTTCTGCACCATCTCCTTGCCCAGGTAGTGGTCGATGCGGTAGATCTGGTCCTCACGGAACAGGGAG[**G563A**]AGATGTGGTTGGACAGCCGG[**T542A**]CAGAGCTCTGCAGGTCCCTCCCGAAGGGCTTCTCCACGATGATGCGGTTCCAGCCTCTGCTGGGAGCCCGGAGCTGCGTTACCCCCTTGAACCCCTCTTCGGGGAGTGAGGATCAGAGCTGCATCATTCAGACGCCCTCCCAGGGGAGTAGAGGCCAGAACCTCCTCGCCCCCGTGGCCACCTCCCGTGCCTTGTGTTCCCAGATGACCCTCTGGCTCAACACCTTCCGGGAAGCCTTCCCACCCTGGGTGCCAGGGTGGCATTGCTGGCATGCTGCCGGGGGCCCGTTCCCCTCCTGCCTCATGCTGGGTCCCCGGTGGGTCTGAGTGGCCTGAAGGCCTGTAGGGGAGCAGGGGGAGGAGGCATCCAAGCCATGGCTTCCTCATTGGTTTTGAAAATGAGAAGAGGACCTTTGCTTTACTACCCCCGCATTCAAAACCAGCCAGAGGACAAGAGCCTCTGGCTGGGTTGGGAAACCACCCAGCGCGGGCCATGCTGCATTCGCAGAGCAAGGCTGCCACCCTGCGGCCTGGCCGGGCCTTTGGGGAAGCAGAGCGGAAAGGCGGTGTTTCGTGGAGCAACGCTGCCACCTTGTGGTCCCGCTGGGGATGGCCCCGGCACCATGGATGCTGCCCAGATCCCCGGCCCCGGACACGCTCATAGAGTGGTGGGAGCACTGCCTGGGCCAGCCTGGCAGGCGGGAAGGGAGGGCAACGGCAAGCCTTACATCTGGCTCATGCAGGACTCGTGAATGTTCTTGGTGACGGCCTCGTAGACGGTCGGGGGCAAGGCCAGGTAGAAGA[**G409A**]GCGGTTGGCCTGTGACCCCAGGTGGAGGGCAT[**T376C**]CATGTGGCTGTTGAGGCGCTGGTAGGAGGCTGCATCATCGTACTGGCCAGCCACATAGGAGTTGCGGGCAAAGAAGTCCTCCAGCTTGAGCTTCTCCTCTGGGGTGGCCTGGGAGACACGGACAGACAGACACACAGACAGATGTCAGCCCCTCTCTTTGAGTCCGTGTGTGTTCTGCCCCAGGGGAGTGGAGGGTCTTCCCTGGAGGTCCAGGGAGGGTGCCCTCAGAAGTCAGTGTCCCCGTCCCACACTGGGTTCAGCCCCATCTTAGCAGCTCTGCACATCCAGAGGGAGGGAGGCCAAAGCAGGCAGCACAGACACTGCCCCAGGCTGGGACCCCTGTGCCTTAGGACAGAGGCCAGATTTCAGGATATTTTGACCTGGGAGAAATACACTGGAGAAAGCTCTCTCTCCAAAATCATGACACCCAACTATGATTGGCGGAGAAAACGCAGCAGAGCACAGCAGGAGGGGACCTGTGGGTCCTGGTCACGGGGGCTGGTAATGGGGGTCTCAAGGAAGTACGAGAGCAGGCGGGGCGGGGCAGGAGAGGAGGAGAGCATCCCGGGATGGGATGGGGGGAGGTCCCCGAAGCTGGCCATGCTGGGGGCTGGTAGAGAGGGCAGAACCAGGCTGGGGGAGGCCCTGACACCACCCACCTTGAAGAAGGGCTCACTCTGTTTGCGGATGTCAGCCACTGTGAGGCGGGAACGGGCATAGCCCA[**C202T**]GATGAAGGTGTTTTCG[**G185T**]GCAGAAGGCCATCCCGGAACAGCCACCTGAGGGCAGGGCACAGCTGTAACCAGTGCGGGCAGGGCAGGACCAGGCCTGTCCCTGGCGGGAGGTCACAGGGGCAGTGGTGGGACACACTTACCAGATGGTG

**Supplementary Figure 2 SNaPshot genotyping profiles for the analyzed *G6PD* variants.** (I) Electropherograms obtained from singleplex SNaPshot reactions for each variant. (II) Multiplex SNaPshot assay results showing representative genotypes of (a) Wild-type for all variants, (b) Heterozygous for G202A and A376G, (c) Homozygous for G202A and A376G, (d) Heterozygous for G844C and (e) Synthetic gBlock control carrying homozygous mutant alleles for all seven *G6PD* variants.


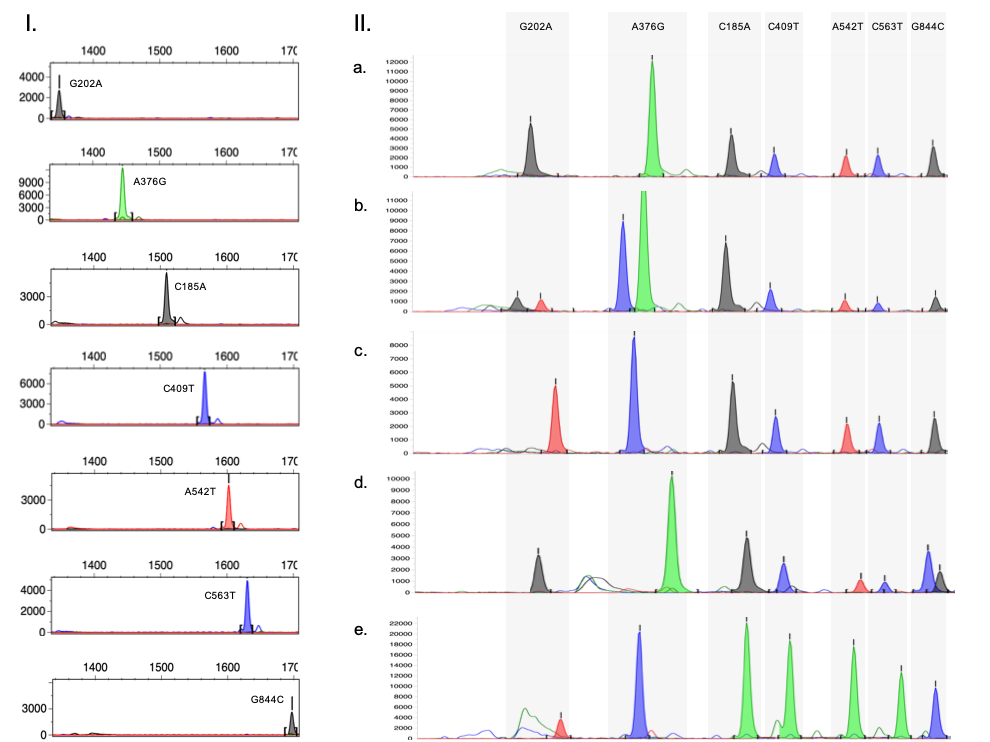


**Supplementary Figure 3** **Linkage Disequilibrium (LD) plots of the analyzed *G6PD* SNPs.** Panel (a) Represents the whole study population, (b) The Manaus population, and (c) The Porto Velho population. Red shading indicates SNP pairs with high linkage disequilibrium. The values shown within each cell correspond to the *r^2^* statistic. LD plots were generated using Haploview software (version 4.2).


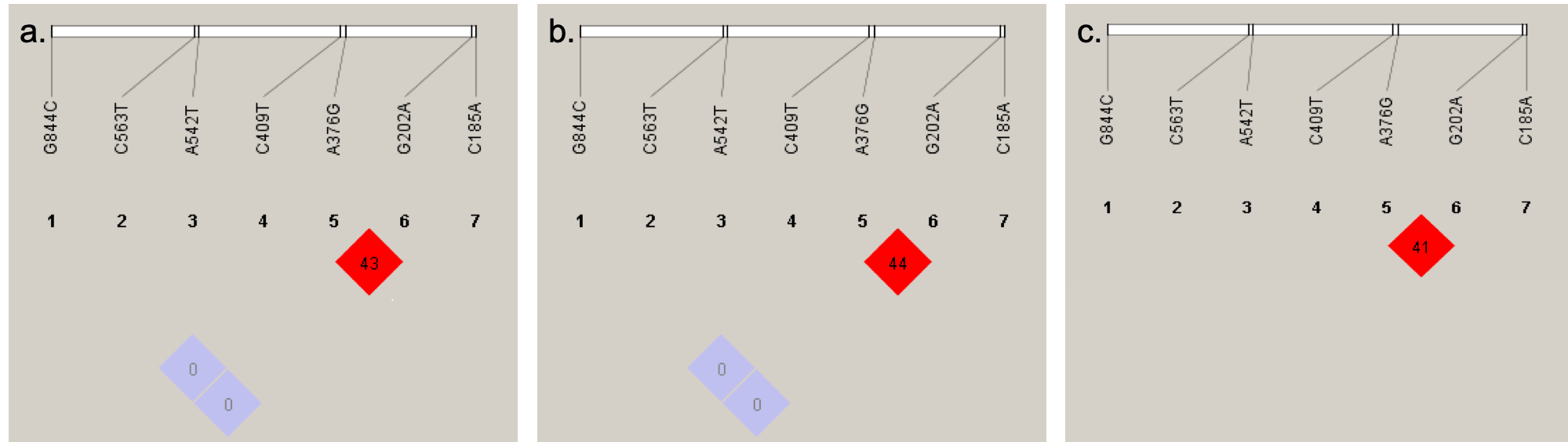

Supplement: Supplementary file 1 [file mmc1.docx]
